# Characterization of the Blood–Brain Barrier Integrity and the Brain Transport of SN-38 in an Orthotopic Xenograft Rat Model of Diffuse Intrinsic Pontine Glioma

**DOI:** 10.3390/pharmaceutics12050399

**Published:** 2020-04-27

**Authors:** Catarina Chaves, Xavier Declèves, Meryam Taghi, Marie-Claude Menet, Joelle Lacombe, Pascale Varlet, Nagore G. Olaciregui, Angel M. Carcaboso, Salvatore Cisternino

**Affiliations:** 1 Variabilité de RéPonse aux Psychotropes, INSERM, U1144, F-75006 Paris, France; catarinachaves1987@gmail.com (C.C.); xavier.decleves@parisdescartes.fr (X.D.); meryam.taghi@parisdescartes.fr (M.T.); marie-claude.menet@parisdescartes.fr (M.-C.M.); 2 Université Paris Descartes, UMR-S 1144, F-75006 Paris, France; 3 Université Paris Diderot, UMR-S 1144, F-75013 Paris, France; 4 Hôpital Universitaire Cochin, Assistance Publique Hôpitaux de Paris, AP-HP, F-75014 Paris, France; 5 Department of Neuropathology, Hôpital Sainte-Anne, Université Paris Descartes, F-75014 Paris, France; j.lacombe@ch-sainte-anne.fr (J.L.); p.varlet@ch-sainte-anne.fr (P.V.); 6 Institut de Recerca, Sant Joan de Déu Hospital, 08950 Barcelona, Spain; ngene@fsjd.org (N.G.O.); amontero@fsjd.org (A.M.C.); 7 Hôpital Universitaire Necker-Enfants Malades, Assistance Publique Hôpitaux de Paris, AP-HP, F-75015 Paris, France

**Keywords:** ATP-binding cassette transporters, biological transporters, blood–brain barrier, camptothecin, glioma, rare disease

## Abstract

The blood–brain barrier (BBB) hinders the brain delivery of many anticancer drugs. In pediatric patients, diffuse intrinsic pontine glioma (DIPG) represents the main cause of brain cancer mortality lacking effective drug therapy. Using sham and DIPG-bearing rats, we analyzed (1) the brain distribution of 3-kDa-Texas red-dextran (TRD) or [^14^C]-sucrose as measures of BBB integrity, and (2) the role of major ATP-binding cassette (ABC) transporters at the BBB on the efflux of the irinotecan metabolite [^3^H]-SN-38. The unaffected [^14^C]-sucrose or TRD distribution in the cerebrum, cerebellum, and brainstem regions in DIPG-bearing animals suggests an intact BBB. Targeted proteomics retrieved no change in P-glycoprotein (P-gp), BCRP, MRP1, and MRP4 levels in the analyzed regions of DIPG rats. In vitro, DIPG cells express BCRP but not P-gp, MRP1, or MRP4. Dual inhibition of P-gp/Bcrp, or Mrp showed a significant increase on SN-38 BBB transport: Cerebrum (8.3-fold and 3-fold, respectively), cerebellum (4.2-fold and 2.8-fold), and brainstem (2.6-fold and 2.2-fold). Elacridar increased [^3^H]-SN-38 brain delivery beyond a P-gp/Bcrp inhibitor effect alone, emphasizing the role of another unidentified transporter in BBB efflux of SN-38. These results confirm a well-preserved BBB in DIPG-bearing rats, along with functional ABC-transporter expression. The development of chemotherapeutic strategies to circumvent ABC-mediated BBB efflux are needed to improve anticancer drug delivery against DIPG.

## 1. Introduction

Diffuse intrinsic pontine glioma (DIPG) is a devastating pediatric cancer that primarily affects the brainstem, representing the leading cause of pediatric brain tumor mortality. DIPG is typically diagnosed in 5- to 10-year-old children, with a rapid onset of symptoms, and less than 20% survive longer than 2 years after diagnosis [1]. Biologically distinct from other adult high-grade gliomas (HGG), DIPG remains untreatable: Tumor-removal surgery cannot be performed due to both its poorly accessible anatomical location and its very diffuse disposition. Radiotherapy alone or in combination with any sort of chemotherapeutic agent has not been proven to increase the average life expectancy of patients or to alter the overall survival in children diagnosed with DIPG [2,3].

As similarly described for other brain tumors, the most likely cause of chemotherapeutic unsuccess in DIPG is related to its location in areas protected by the blood–brain barrier (BBB), which represents a physical and biochemical hindrance to the tissue delivery of systemically administered drugs [4,5,6]. The BBB properties within the DIPG-affected CNS regions, namely the cortex and brainstem, have been poorly addressed, which may represent a further difficulty in the establishment of optimized therapeutic strategies for DIPG. DIPG tumor cells may proliferate and migrate without disrupting the anatomical structures until very late in the disease, allowing conservation of the BBB integrity in patients [2,7,8]. In addition to this physical BBB diffusion impediment of drugs from the blood to the nerve parenchyma, drug efflux transporters present at the BBB, such as the P-glycoprotein (P-gp, ABCB1), the breast cancer resistance protein (BCRP, ABCG2), and multidrug resistance proteins (MRPs, ABCCs), can profoundly restrict the brain parenchyma penetration of numerous anticancer drugs and considerably limit the chemotherapy efficacy in patients [5,9,10]. The recent development of a preclinical orthotopic DIPG model, through the establishment of the HSJD-DIPG-007 tumor cell line of human origin [11], has both enabled the screening of active candidate drugs in vitro, such as the camptothecin SN-38, the active metabolite of irinotecan, and the development of a reliable and clinically relevant DIPG rat model. Irinotecan, in particular, has been used as an agent in adjuvant chemotherapeutic combinations for the treatment of pediatric brain tumors, including DIPG, and is currently part of ongoing clinical trials (clinicaltrials.gov id: NCT00890786; id: NCT01837862; id: NCT03086616).

The present study dedicated its focus to the biochemical and functional analysis of the BBB properties in control and patient-derived DIPG xenografted rats in three critical brain regions: Cortex, cerebellum, and brainstem. The attention is particularly focused on SN-38 carrier-mediated transport in selected CNS structures, such as the brainstem and the cerebellum. These regions are known to be characteristically affected in patients with DIPG, and the implication of ABC transporters prominently expressed at the BBB (e.g., P-gp, BCRP, and MRPs) in the brain efflux transport of SN-38 was assessed. Therefore, we 1) analyzed the BBB integrity and the expression of several BBB markers in control and DIPG-bearing animals, and 2) characterized the BBB transport of SN-38 into the cerebellum, brainstem, and cerebrum and its ABC transporter-driven efflux, and how the DIPG condition and transporter inhibitors may affect such transport.

In DIPG rats, the tight junctions and ABC efflux transporters (P-gp, Bcrp, Mrps) are preserved. DIPG cells express BCRP but not P-gp and MRPs. The BBB therefore constitutes a major obstacle to the access of drugs to the brain parenchyma. Elacridar, a dual P-gp and Bcrp inhibitor used in clinical trials [12,13], in our study, revealed an unrelated P-gp/Bcrp-mediated SN-38 cerebrum efflux. The combination of P-gp, Bcrp, and Mrp inhibitions was found to be more effective at increasing the SN-38 distribution into the brain. These results allow a better understanding of the mechanisms hampering efficient drug delivery within the tumor site and their effectiveness, opening a new horizon for the exploration of innovative strategies that may combine SN-38, ABC inhibitors, or other novel therapeutic agents as advanced delivery systems for the treatment of DIPG. 

## 2. Materials and Methods

### 2.1. Primary DIPG Cell Culture

The HSJD-DIPG-007 cell line was established from DIPG tumor tissue issued at the Sant Joan de Déu Hospital (Barcelona, Spain). These cells express archetypal histone mutations H3F31 (H3.3), K27M-mutated protein (H3K27M), and the activin A receptor type 1 (ACVR1) R206H mutation frequently observed in patients with DIPG [14,15]. Cells were cultured in serum-free DMEM-F12/Neurobasal-A (1:1, Gibco, Courtaboeuf, France), supplemented with HEPES (10 mM, Gibco), sodium pyruvate (1 mM, Gibco), non-essential amino acids (Gibco), glutaMAX-I supplement (Gibco), antibiotic-antimycotic (100 U mL^−1^ penicillin, 100 µg mL^–1^ streptomycin, 0.25 µg mL^−1^ amphotericin B, Life Technologies, Courtaboeuf, France), B-27 supplement (vitamin A), human EGF (20 ng mL^−1^, Peprotech, Neuilly-sur-Seine, France), human bFGF (20 ng mL^−1^, Peprotech), human PDGF-AA (10 ng mL^−1^, Peprotech), human PDGF-BB (10 ng mL^−1^, Peprotech), and heparin (2 µg mL^−1^, Sigma, St Quentin, France).

### 2.2. Animals

All animal experiments complied with the standards and guidelines promulgated by the latest European Union Council Directive (2010/63/EU) and were approved by the ethics review committee of Paris Descartes University and the French ministry, APAFIS#6656-20l6042911227858, 28th March 2017).

Four-week-old (w.o.) athymic RH-Foxn1rnu female nude rats (100–120 g) (Envigo Laboratories, Gannat, France) were maintained under standard 12-h light/dark conditions, in a temperature and humidity-controlled facility under pathogen-free conditions in air-filtered cages. In a preliminary experiment, male and female rats were used to ascertain sex differences. The triple knock-out (TKO) strain (Abcb1a(−/−), Abcb1b(−/−), Abcg2(−/−)) was bred in house from mice progenitors obtained from the A.H. Schinkel team (Amsterdam, Netherlands). This strain derived from Fvb mice. Female wild-type (WT; Janvier, France) and female TKO (25 ± 5 g; 9 ± 2 w.o.) were housed in a controlled environment (22 ± 3 °C; 55 ± 10% humidity) under a 12-h light cycle. Animals had access to food and water ad libitum. All animals were acclimated for a minimum of 7 days prior to use in experiments.

### 2.3. Stereotactic Transplantation of DIPG cells

Four-week-old athymic RH-Foxn1rnu nude rats (100–120g) were subjected to DIPG cell transplantation, in accordance with approved institutional animal use and care protocols.

Prior to surgery, rats were anesthetized by intraperitoneal (i.p) injection of a ketamine-xylazine mixture (90–10 mg kg^−1^), and placed in a stereotactic apparatus. The skull of the rat was exposed to reveal lambda and bregma and, following the accurate coordinates calculated from the lambda position, a small opening was made using a drill appropriately assembled to the stereotaxic apparatus. HSJD-DIPG-007 cells were injected into the IVth ventricle (8.0 mm depth from the surface of the exposed skull) with a 26-gauge Hamilton syringe (Dutscher, Brumath, France). In total, 750,000 live DIPG tumor cells contained in a 7.5-μL volume were injected into the IVth ventricle. Animals were monitored daily after surgery.

Sham surgeries were also performed in order to monitor if the eventual differential results obtained in the DIPG groups were due to the disease model itself or to the performed invasive surgery.

### 2.4. Immunohistochemistry Analyses

DIPG rat xenografts were exsanguinated by intracardiac perfusion (25 mL·min^−1^) of PBS with subsequent perfusion of a zinc formalin solution (Sigma) for brain tissue fixation. Extracted brains were formalin-fixed for 24 h prior to paraffin-embedding and sectioning using a sliding microtome (3-µm sections). Formalin-fixed sections were then subjected to either hematoxylin phloxine saffron (HPS) staining or immunohistochemical (IHC) staining.

HPS staining was run on a Tissue-Tek^®^ Prisma™ automated slide stainer (Sakura Finetek^®^, Villeneuve d’Ascq, France), while IHC was done on the automated Discovery^®^ XT slide staining system (Ventana Medical system^®^, Illkirch, France). In brief, sections were deparaffinized and microwave antigen retrieval was performed using the semi-automatized MicroMED T/T Mega system (Hacker Instruments, Winnsboro, SC, USA) for 30 min at 98 °C for the H3K27M rabbit polyclonal antibody (1/1000, Millipore Cat# ABE419, RRID:AB_2728728). Staining used the RTU Vectastain Universal detection system (AB_2336829; Vector laboratories, Burligame, CA, USA). Slides were then scanned on a NanoZoomer Digital Slide Scanner (Hamamatsu, Japan).

### 2.5. Radiolabeled Compounds and Chemicals

Synthesis of [^3^H]-SN-38 (0.629 TBq mmol^–1^) was performed by Moravek Inc (Brea, CA, USA). [^14^C]-sucrose (0.0215 TBq mmol^−1^), and [^3^H]-diazepam (3.3 TBq mmol^−1^) were purchased from Perkin Elmer Life sciences (Villebon, France). Elacridar (also known as GF120918), MK571, Ko143, and valspodar (also known as PSC833) were purchased from Sigma.

### 2.6. In Situ Carotid Perfusion

#### 2.6.1. Surgical Procedure and Perfusion

Transport of [^3^H]-SN-38 (~30 nmol L^−1^) at the luminal side of the BBB was measured by in situ brain perfusion in 8-week-old rats [16,17]. Using this technique, the vascular composition of the brain is totally substituted by an artificial fluid whose constitution can be modified. Rats or mice were anesthetized by i.p. administration of a ketamine-xylazine mixture (rats 90–10 mg kg^−1^, mice 140–8 mg kg^−1^). Briefly, a catheter was inserted into the right carotid artery after ligation of the appropriate vessels. The heart was removed, and carotid perfusion started immediately at a constant flow rate of 10 or 2.5 mL min^−1^, in the rat and mouse, respectively. Each animal was perfused with [^3^H]-SN-38 (0.011 MBq mL^−1^) or [^3^H]-diazepam (0.01 MBq mL^−1^), and a vascular marker [^14^C]-sucrose (0.0037 MBq mL^−1^), in the presence or absence of different ABC transporter inhibitors (Elacridar 10 µM; Valspodar 10 µM; Ko143 10 µM; MK571 100 µM). Perfusion was terminated by decapitating the animal at 120 s except for [^3^H]-diazepam (60 s). The brain and pons were removed from the skull. The dissected tissues and aliquots of perfusion fluid were weighed. All samples were treated with Solvable^®^ (Perkin Elmer) for 24 h, and then mixed with Ultima-gold XR^®^ (Perkin Elmer). The radioactive counting was performed using a Perkin Elmer Tri-Carb counter to measure ^3^H and ^14^C disintegrations per minute (dpm) for each sample.

#### 2.6.2. Perfusion Fluid

The perfusion fluid consisted of Krebs physiological saline buffered with carbonates (mmol L^−1^): 128 NaCl, 4.2 KCl, 1.5 CaCl_2_, 24 NaHCO_3_, 2.4 NaH_2_PO_4_, 0.9 MgSO_4_, and 9 D-glucose, warmed to 37 °C and gassed with 95% O_2_/5% CO_2_ to bring the pH to 7.40. The pH of the perfusion fluid was checked with a digital pH-meter (± 0.05 pH-units) right before perfusion.

#### 2.6.3. Apparent Initial Tissue Distribution Volume and Transport Parameters

All the pharmacokinetic parameter calculations were performed as described previously [16]. Briefly, the vascular volume (Vv; µL g^−1^), also used as a BBB integrity marker, was estimated based on the tissue distribution of [^14^C]-sucrose calculated by Equation (1):

(1) $$Vv=\frac{X^{*}}{C_{perf}^{*}},$$

where X* (dpm·g^−1^) is the quantity of [^14^C]-sucrose found in the tissue, and C*_perf_ (dpm·µL^−1^) represents the [^14^C]-sucrose concentration in the perfusion fluid. The physiological cerebrum Vv value was reported [18].

The apparent [^3^H]-SN-38 tissue distribution volume (Vtissue, µL g^−1^) was calculated as:

(2) $$Vtissue=\frac{Xtissue}{Cperf},$$

where Xtissue (dpm g^−^^1^) is the calculated brain amount of [3H]-SN-38 and Cperf (dpm µL^−^^1^) its concentration in the perfusion fluid:

(3) $$X_{tissue}=X_{tot}-V_{v}C_{perf},$$

where Xtot (dpm g^−1^) is the total quantity of [^3^H]-SN-38 measured in the sample tissue. The amount of [^3^H]-SN-38 in the vascular “[^14^C]-sucrose” space (*V_v_C_perf_*) was calculated and subtracted from the total (Xtot) (Equation (3)). 

The initial transport rate, also called the brain/tissue clearance, and corrected from the vascular contamination is expressed as a Kin (µL s^−1^ g^−1^) parameter and was calculated from:

(4) $$K_{in}=\frac{V_{tissue}}{T},$$

where T is the perfusion time (s).

The selected time of 120 s ensured that the distribution was in the linear part of the apparent [^3^H]-SN-38 tissue distribution (data not shown).

Tissue extraction E (%) is given by:

(5) $$E_{\%}=\frac{K_{in}}{F}\cdot 100,$$

where F (µL s^−1^ g^−1^) is the perfusion flow measured with [^3^H]-diazepam, a 100% extracted compound [16].

### 2.7. Injection of Texas Red Dextran, Tissue Processing, and Confocal Imaging of Brain Cryostections

Texas Red-conjugated 3kDa dextran (TRD) (ThermoFisher) was used to evaluate the BBB integrity in DIPG xenografts [10] in comparison to the control (sham-operated animals). Animals were anesthetized with ketamine and xylazine (90 and 10 mg kg^−1^, respectively) prior to a 6 mg kg^−1^ TRD intravenous injection into the tail vein. TRD was allowed to circulate for 10 min, animals were euthanized, and brains were immediately removed. Brains were fixed by immersion in 4% paraformaldehyde (PFA) for 24 h, dehydrated by immersion in 20% sucrose for another 24 h, and subsequently flash-frozen in isopentane and stored at −80 °C. Sagittal brain sections (40-µm thick) were then obtained with a cryostat (Leica, Nanterre, France). Sections were then incubated with blocking solution (10% goat serum, 0.1% Triton X-100 in PBS) for 30 min at room temperature (RT), and with the primary mouse IgG1 anti-human nuclei antibody (1:250, Millipore Cat# MAB4383, RRID: AB_827439). Sections were then incubated with goat anti-rabbit AlexaFluor 633-conjugated (AB_2535731) and goat anti-mouse IgG1 AlexaFluor 488-conjugated (AB_2556548) secondary antibodies (1:500, 1.5 h at RT, Thermo Fisher Scientific), and nuclei were counterstained with Hoechst 33342. Images (1024 × 1024 pixels) were recorded on a confocal microscope (TCS-SP2; Leica) equipped with an X40 oil-immersion objective (NA = 1.00). The pixel physical size was 378 nm as the electronic zoom value was fixed for all acquisitions, while the diffraction-limited resolution was 345 nm according to Rayleigh criteria at 565 nm.

### 2.8. Brain Protein Extraction, Digestion, and Quantification by UHPLC-MS/MS

Cortex, cerebellum, and brainstem isolation and crude membrane fraction extraction: Cell crude membrane fractions were isolated as described previously with minor modifications [19]. Brains were extracted from control rats (sham-operated) and DIPG rat xenografts, and brain cortices, cerebella, and brainstems were collected separately in isotonic 20 mM phosphate buffer pH 7.4, 0.1 M KCl. These isolates were washed at least twice in buffer A containing a protease inhibitor cocktail, minced into 1-mm pieces, and homogenized by using an Ultraturrax^®^ (IKA, Lille, France) for 5 min at 4 °C. The obtained homogenates were further submitted to sonication using a BioRuptor (Diagenode, Seraing, Ougrée, Belgium) for 5 min at high frequency. The tissue lysates were centrifuged at 12,800 g for 15 min at 4 °C, supernatants collected, and ultracentrifuged at 100,000 g for 60 min at 4 °C. The obtained pellets were resuspended in a 20 mM Tris pH 7.4, 0.25 M sucrose, 5.4 mM EDTA buffer containing protease inhibitor cocktail.

Protein digestion: Crude membrane fractions were digested as previously described [20,21,22]. In brief, 50 μg of proteins were first solubilized in denaturing buffer (7 M guanidine hydrochloride, 10 mM EDTA, 500 mM Tris pH 8.5), and then reduced by dithiotreitol (DTT) and alkylated by iodoacetamide. The protein samples were subsequently precipitated in a methanol-chloroforme-water mixture and resolubilized in 1.2 M urea, 0.1 M Tris pH 8.5. The resulting proteins were first digested using rLysC endoprotease (enzyme:protein ratio = 1:50) for 3 h at RT. A second protein digestion using trypsin (enzyme:protein ratio = 1:100) and 0.05% (*w*/*w*) ProteaseMAX^®^ was performed as samples were incubated at 37 °C overnight. Prior to UHPLC-MS/MS analysis, the stable isotope-labeled peptide mixture (750 fmol of each labeled peptide) was added to the trypsic digests including 100 fmol L^−1^ of six synthetic *E. coli* control peptides used in the evaluation of the reproducibility of peptide LC-MS/MS analysis (Hi3 Ecoli^®^ standards, Waters, Guyancourt, France). Samples were dried using a centrifugal vacuum concentrator (Maxi-Dry Lyo; Heto Lab Equipment, Allerod, Denmark), stored at −80 °C, and solubilized until analysis in a mixture of 10% acetonitrile, 90% water, and 0.1% formic acid. 

In silico selection of proteotypic peptide candidates for P-gp, BCRP, MRP1, MRP4, and Nestin: General criteria relative to stability, compatibility for triple-quadrupole detection, and protein specificity were applied for the selection of peptide candidates obtained from the list of sequences identified in the DDA experiment [23,24]. The Protein Information Resource peptide search was utilized to verify the specificity of each peptide [25]. The peptides hereby used for the quantification of P-gp and BCRP have been previously identified [23,26,27,28]. A list of the proteotypic peptides synthetized in light and heavy forms and used as standards is presented in Table S1 (Supplementary Materials), along with their species specificity.

Absolute protein quantification by UHPLC-MS/MS: Protein quantification (P-gp, BCRP, MRP1, MRP4, Nestin) in analyzed samples was performed using a QTAP approach [22,26], using an ACQUITY UPLC H-Class^®^ system coupled to a Waters Xevo^®^ TQ-S mass spectrometer (Waters) operated in multiple reaction monitoring (MRM) mode. Skyline software (version 3.5.0.9319) was used to export the area ratios of light to labeled peptides and quantification was performed from calibration curves by using GraphPad Prism^®^ 6.0 software (San Diego, CA, USA).

### 2.9. Statistical Analysis

Data were analyzed with GraphPad Prism^®^ 6.0 software. Results are expressed as mean ± SD. The student t-test and one-way ANOVA with Tukey’s multiple comparisons tests were used to compare the different studied groups. Statistical significance was set at *p* < 0.05 for all the tests.

## 3. Results

### 3.1. Establishment of the Experimental Xenograft Rat Model of DIPG

**Figure 1:**
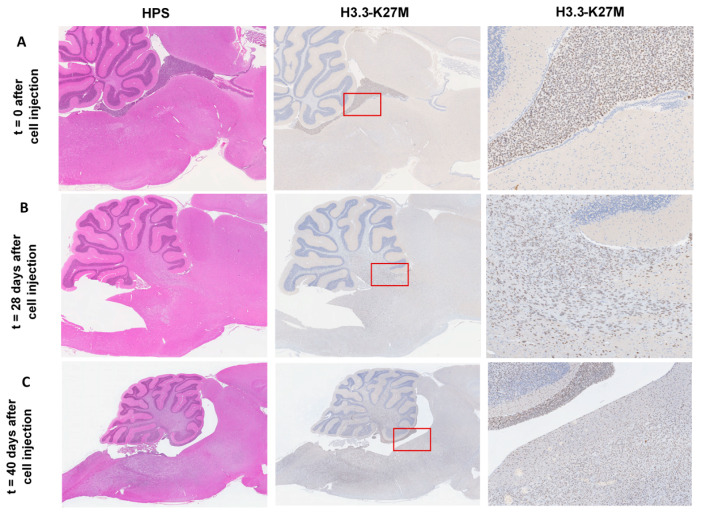
Evaluation of correct diffuse intrinsic pontine glioma (DIPG) cell implantation and tumor development for the establishment of DIPG xenografts in RH-Foxn1rnu nude rats. (**A**) Sagittal rat brain section with HPS (hematoxylin phloxine saffron staining, left panel, 60× magnification) and immunohistochemistry (IHC) staining for H3.3-K27M (middle and right panel, 60× and 400× magnification, respectively, brown nuclei-stained cells) at D0 show correct DIPG cell injection into the 4^th^ ventricle of the rat brain. (**B**) Sagittal rat brain section with HPS (left panel, 60× magnification) and IHC staining for H3.3-K27M (middle and right panel, 60× and 400× magnification, respectively, brown nuclei-stained cells) at D28 show DIPG cells largely diffused into the brainstem and cerebellum 4 weeks after DIPG cell implantation into the 4th ventricle of the rat brain. (**C**) Sagittal rat brain section with HPS (left panel, 40× magnification) and IHC staining for H3.3-K27M (middle and right panel, 40× and 200× magnification, respectively, brown nuclei-stained cells) at D40 show an increasing degree of infiltration of DIPG cells and necrosis of the brain tissue.

In a previous study, the HSJD-DIPG-007 tumor cell line showed the expression of the histone variant H3F31 (H3.3), K27M-mutated protein (H3K27M), and the ACVR1 mutation [14], previously defined as the hallmark mutations in DIPG. A preliminary time course study was conducted to evaluate the accurate implantation of tumor cells into the rat brain 4th ventricle, as well as the tumor growth and extension by IHC. Rats were euthanized at pre-determined time points (D0, D28, and D40), and whole brains were immediately removed, processed, and sliced to obtain serial cross sections in the sagittal plane. IHC sections evidenced a correct injection of cells into the rat brain 4th ventricle at D0 (Figure 1A), through the detection of the H3K27M-mutated histone. By D28, a strong positivity throughout the pons and cerebellum is noticeable (Figure 1B) and with extension over the brainstem, evidencing significant tumor growth and expansion, a time at which animals remain asymptomatic. The onset of symptoms was observed by D35, and by D40 DIPG tumor infiltration shown to have increased considerably, affecting the whole cerebellum and brainstem, and diffusing towards the diencephalon and adjacent brain structures (Figure 1C), accompanied by a severe weight loss (~20%). Thus, a reproducible disease model was established at four weeks after tumor cell implantation, with significant tumor infiltration around the pons and cerebellum and no development of clinical signs or a compromise of the animal well-being. This time-point was chosen for subsequent experiments. To ensure animals were successfully tumor cell-engrafted in each experiment performed four weeks after DIPG cell implantation, a sentinel was randomly chosen after each tumor cell implantation experiment, for histology and IHC purposes afterward. IHC monitoring ensured successful DIPG cell-engraftment, with progressive tumor growth in each time animals were euthanized four weeks post implantation (data not shown).

### 3.2. Blood–Brain Barrier Integrity and ABC Transporters Expression in DIPG

#### 3.2.1. BBB Permeability to [^14^C]-Sucrose and Texas Red-Conjugated Dextran

**Figure 2:**
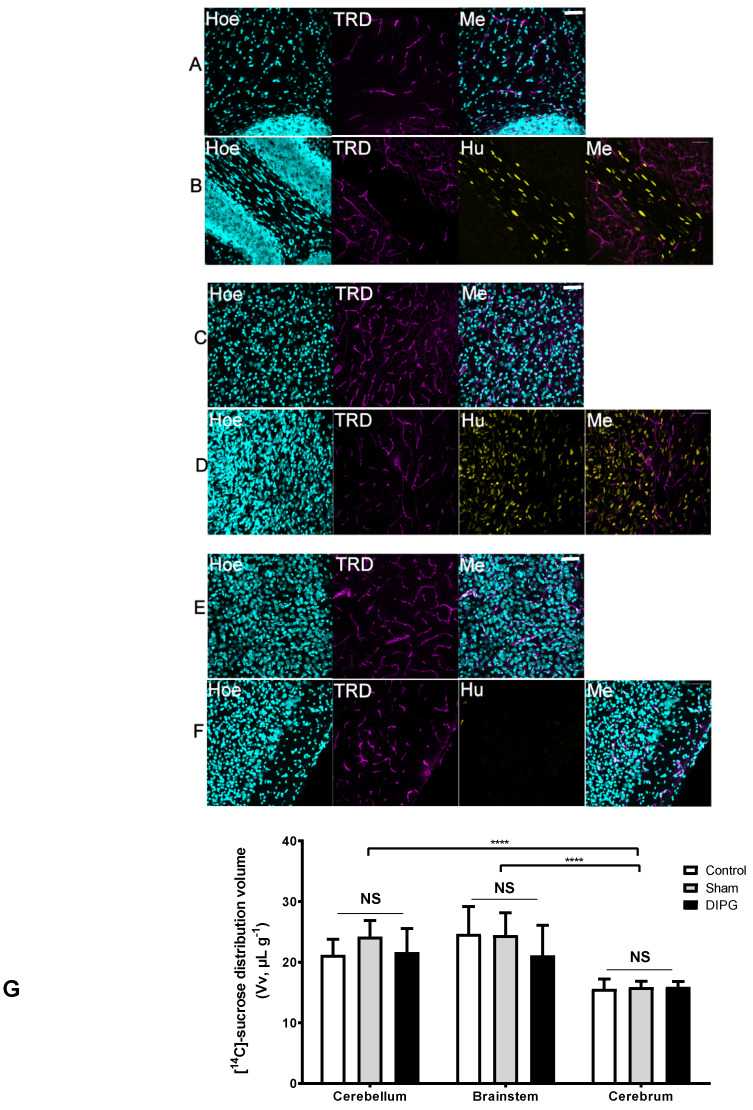
(**A–G**) Effect of the presence of DIPG (patient-derived HSJD-DIPG-007 tumor cells) on the integrity of the BBB to the passage of 3-kDa Texas Red-labeled Dextran (TRD, magenta) in control (**A**, cerebellum; **C**, brainstem; **E**, cortex) and DIPG xenografts (**B**, cerebellum; **D**, brainstem; **F**, cortex), and with [^14^C]-sucrose (**G**, cerebellum, brainstem, and cerebrum). (**A–F**) Sagittal brain sections were counterstained with Hoechst 33342 (Hoe, blue) to reveal the cell nuclei, and with an anti-human nuclei (Hu, yellow) to reveal the presence of tumor cells of human origin (scale bar = 50 µm; Me, merge). DIPG-bearing animals were used 4 weeks after stereotaxic injection of DIPG cells into the IVth ventricle, and DIPG non-bearing animals were used as controls, where the BBB integrity is conserved. The presence of DIPG cells does not disrupt the BBB in either the cerebellum, brainstem, or brain cortex, as it remained impermeable to the passage of 3 kDa Texas red dextran (TRD) from the brain vessels into the brain parenchyma. (**G**) Global chart showing the [^14^C]-sucrose tissue volume (Vv; μL g^−1^) within the cerebellum, brainstem, and cerebrum, measured by in situ brain perfusion in 8-week-old RH-Foxn1rnu nude rats. Results are expressed as mean Vv ± S.D. (µL g^−1^; *n* = 7–10 animals per group). One-way ANOVA was performed to analyze Vv differences between control (non-operated), sham, and DIPG animals, NS = no statistical different between groups. Two-way ANOVA was performed to analyze Vv differences between brain regions, **** *p* < 0.0001 vs. cerebrum.

To characterize the BBB regional integrity by microscopy studies, we injected TRD intravenously in control and DIPG-bearing rats, which is commonly used as a vascular marker in vivo given its large molecular weight of 3 kDa. We subsequently analyzed its fluorescence staining in sagittal brain sections, namely in the cerebellum, brainstem, and brain cortex. The results obtained by confocal microscopy (Figure 2A–F) evidence that in both control and DIPG-bearing rats, inclusively in brain regions affected by the presence of tumor cells, the TRD-derived fluorescence remained within the vascular luminal space, without any diffusion into the extravascular space.

To further evaluate the possible BBB loss of integrity, the [^14^C]-sucrose tissue distribution volume was measured in control and DIPG xenografts (8 weeks old 4 weeks post-implantation). Sucrose is a small molecule (342 g·mol^−1^) commonly used as a sensitive marker of the tissue vascular space and of BBB integrity since it does not cross intact plasma membranes and paracellular diffusion is impeded by tight junctions between brain endothelial cells. A first preliminary experiment conducted in male and female rats disclosed no difference regarding the gender of the animal chosen for the subsequent studies (Supplementary Materials Figure S1). The [^14^C]-sucrose tissue volumes (Vv) of control, sham, or DIPG-bearing rats did not differ significantly, independently of the analyzed brain region (Figure 2G), which points out no loss of the BBB integrity in DIPG. The vascular volume measured for the cerebellum and the brainstem is physiologically higher than that of the cerebrum as previously reported [29]. These elements further confirmed the intact state of the BBB in this DIPG animal model whatever the brain structure of interest, as suggested by the confocal results of the TRD dispersion limited to the brain vasculature.

#### 3.2.2. Expression of Selected Proteins and ABC Transporters by Targeted Proteomics

**Figure 3:**
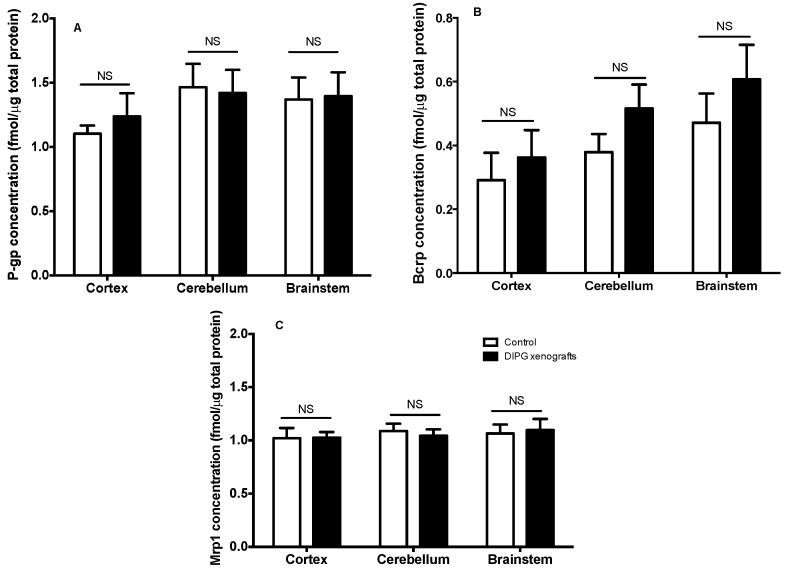
P-glycoprtoein (P-gp) (**A**), Bcrp (**B**), and Mrp1 (**C**) expression levels in whole homogenates from the cortex, cerebellum, and brainstem of control sham vs. DIPG-bearing rats, measured by HPLC-MS/MS of 8-week-old RH-Foxn1rnu nude rats. Results are expressed as the mean absolute concentration (fmol μg^−1^) (*n* = 5 animals per group). Two-way ANOVA, no statistical difference between groups was observed (*p* > 0.05), regardless of the brain structure analyzed for any of the ATP-binding cassette (ABC) transporters measured (i.e. P-gp, Bcrp, Mrp1).

We further analyzed the consequences of DIPG tumor growth and infiltration on the expression of several proteins, such as ABC transporters, with some known (i.e., P-gp) to be only expressed at the BBB. UHPLC–MS/MS analyses were carried to follow such eventual variation on the levels of expression of ABC transporters in DIPG xenografts in comparison to control animals, using proteins extracted from the total brain cortex, brainstem, and cerebellum homogenates. The obtained results, shown in Figure 3, demonstrate that the expression of P-gp, Bcrp, and Mrp1 was not modified in the DIPG condition, regardless of the brain region analyzed, while the levels of Mrp4 in these samples proved to be below the limit of quantification (LOQ). Indeed, the low amount of tissue material (e.g., brainstem) impeded the ability to isolate microvessels, making the protein quantification achievable only on whole tissue homogenates. A trend for an increased overall Bcrp level is seen in the three brain structures of DIPG-bearing xenografts in comparison to control animals, although it did not reach statistical significance. This finding is most likely due to the presence of human BCRP in DIPG cells alone (see Table 1) also quantified in the structures as the performed UHPLC-MS-MS method does not distinguish rat from human BCRP. This result could suggest the progression of the tumor in the cortex, brainstem, and cerebellum at this stage. Additionally, the quantification of nestin, a cell surface marker used as a biomarker for glioma stem cells (Table 1), further revealed the presence and spreading of the tumor within these regions in the DIPG group, while no nestin was detected in the control group (data not shown).

### 3.3. Initial BBB Transport Rate and Role of Efflux Transporters of [^3^H]-SN-38 at the Rat Cerebrum, Cerebellum, and Brainstem

**Figure 4:**
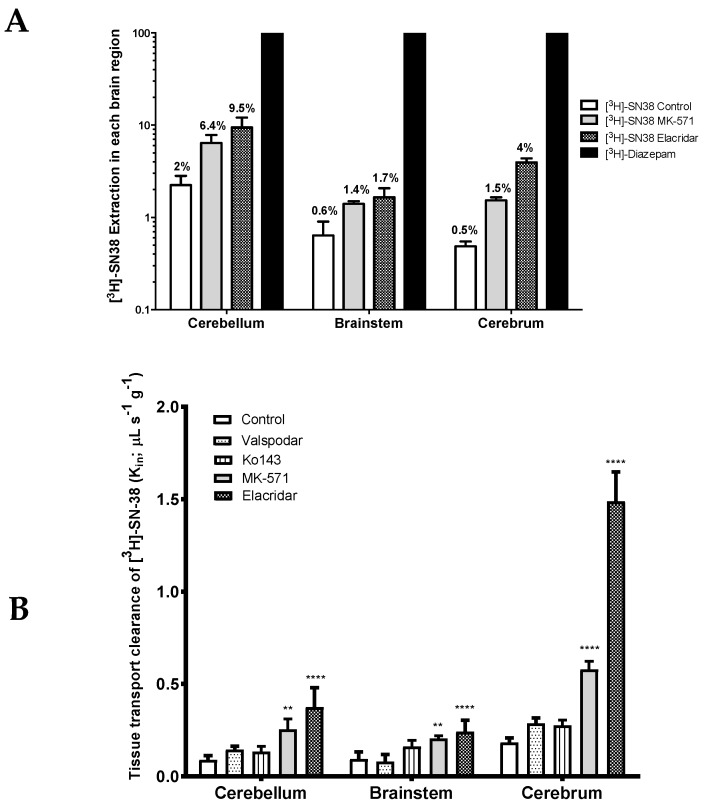
(**A**) Tissue extraction of [^3^H]-SN-38 (E; %) expressed as the percent of [^3^H]-diazepam brain transport (set to 100% in every analyzed region). Results are expressed as mean ± S.D. (*n* = 5 rats per group). (**B**) Intrinsic tissue transport clearance (Kin; μL s^−1^ g^−1^) of [^3^H]-SN-38 within the cerebellum, brainstem, and cerebrum, in the presence or absence of ABC transporter(s) inhibitor measured by in situ brain perfusion in 8-week-old RH-Foxn1rnu nude rats. Results are expressed as mean ± S.D. (*n* = 5 animals per group). One-way ANOVA, ** *p* < 0.01; **** *p* < 0.0001 vs. control group.

RH-Foxn1rnu nude rats (8 weeks old) were in situ brain perfused with [^3^H]-SN-38 for further characterization of its initial brain transport clearance (Kin; µL s^−1^ g^−1^) and assessment of the ABC transporter role at the luminal BBB. The tissue clearance of in situ brain perfused with [^3^H]-SN-38 was compared against the reference [^3^H]-diazepam, a 100% BBB extracted compound used as a flow marker [17]. Differences between the vascular flow as measured with diazepam amongst these structures are known and agree with the literature [30]. The tissue extraction of [^3^H]-SN-38 was about 2% in the cerebellum, 0.6% in the brainstem, and 0.5% in the cerebrum (Figure 4A). Such results underline the relatively poor intrinsic BBB permeability of SN-38 into these tissues. As its extraction is below 30%, the tissue distribution is termed flow independent, Thus, any variation in vascular/blood flow would have no significant impact on its distribution into these tissues. However, carrier-mediated mechanisms could represent critical factors in the SN-38 permeability.

ABC transporter inhibitors were used and added to the perfusion fluid in order to explore the implication of several ABC transporters in the efflux of [^3^H]-SN-38 at the BBB of different CNS regions: Cerebrum, cerebellum, and brainstem.

P-gp inhibition alone using the selective P-gp inhibitor valspodar (PSC-833) did not contribute to a significant increase in the BBB permeability of [^3^H]-SN-38 in the cerebrum, or in any of the other analyzed brain areas (Figure 4B). Similar results were obtained when Bcrp was inhibited alone using the highly selective Bcrp inhibitor Ko143. Nonetheless, dual inhibition of P-gp and Bcrp with elacridar greatly increased the BBB permeability of [^3^H]-SN-38 in all analyzed structures, namely in the cerebrum (8.3-fold), cerebellum (4.2-fold), and brainstem (2.6-fold) (*p* < 0.0001) (Figure 4A,B). Given the considerably greater effect of elacridar when compared to the effects of valspodar and Ko143 alone, or even of the sum of their individual effects, on the BBB permeability of [^3^H]-SN-38, we cannot rule out the (1) elacridar-driven inhibition of other ABC transporters yet to be described to participate in the efflux of SN-38, and/or (2) a synergistic activity between P-gp and Bcrp in the efflux of [^3^H]-SN-38 at the BBB. Furthermore, these results suggest that both P-gp and Bcrp might play a significant role in limiting the BBB permeability of their substrates within the rat cerebrum, but their importance cannot be neglected in other brain regions, such as in the brainstem and the cerebellum.

The inhibition of transporters from the MRP subfamily by the co-perfusion of [^3^H]-SN-38 together with the MRP non-selective inhibitor MK-571 resulted in a significant increase in the BBB permeability to [^3^H]-SN-38. The greatest increase in the [^3^H]-SN-38 BBB permeability was observed within the cerebrum (3.0-fold increase vs. control), followed by the cerebellum (2.8-fold) and brainstem (2.2-fold) (Figure 4A,B). These results support that transporters of the MRP subfamily present at the BBB may contribute to the brain-to-blood efflux of [^3^H]-SN-38.

### 3.4. Cerebrum, Cerebellum, and Brainstem BBB Mouse Transport of [^3^H]-SN-38: Role of ABC Transporters

In order to explore whether the same extent of brain transport and ABC transporter-mediated efflux of [^3^H]-SN-38 is found in the mouse BBB, further studies using in situ brain perfusion of radiolabeled [^3^H]-SN-38, and [^14^C]-sucrose were conducted using WT and TKO (Abcb1a(−/−), Abcb1b(−/−), Abcg2(−/−)) Fvb mice.

**Figure 5:**
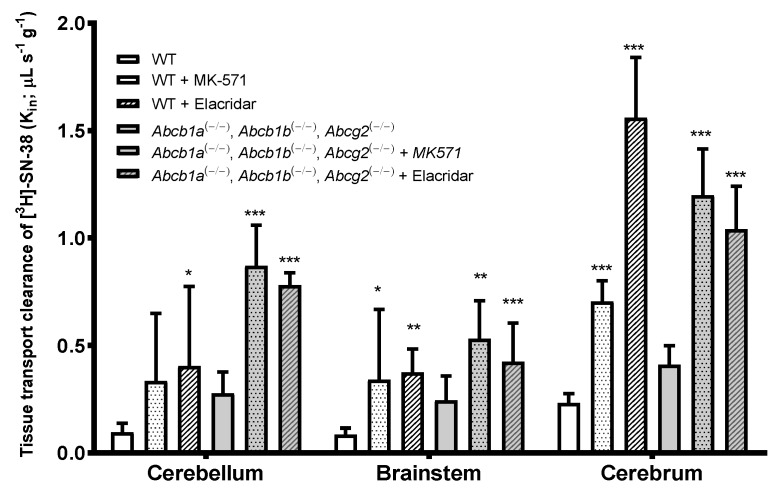
Brain transport (Kin; μL s^−1^ g^−1^) of [^3^H]-SN-38 with and without co-perfusion of the dual P-gp/Bcrp inhibitor Elacridar (10 μM) or Mrp inhibitor MK-571 (100 μM), measured by in situ brain perfusion in wild-type (WT) and triple knock-out (TKO) (Abcb1a(−/−), Abcb1b(−/−), Abcg2(−/−)) mice. Results are expressed as mean ± S.D. (*n* = 4–6 mice per group). One-way ANOVA, * *p* < 0.05; ** *p* < 0.01; *** *p* < 0.001 vs. control group.

TKO (Abcb1a(−/−), Abcb1b(−/−), Abcg2(−/−)) Fvb mice showed an increase in the BBB permeability to [^3^H]-SN-38 in the cerebellum, brainstem, and cerebrum, which was more prominent in the cerebrum. However, it did not result in a statistically significant increase when compared to WT mice (1.8-fold, *p* > 0.05) (Figure 5). This suggests a weak-to-moderate role of P-gp and Bcrp on the BBB permeability of [^3^H]-SN-38 in mice or the contribution of an additive mechanism that functionally compensates for the efflux.

The co-perfusion of [^3^H]-SN-38 with the P-gp/Bcrp dual inhibitor, elacridar, evidenced that the tissue clearance of [^3^H]-SN-38 in the different brain regions analyzed was clearly distinct from the one found in TKO mice. While in TKO mice, the Kin of [^3^H]-SN-38 in the brainstem and cerebrum was not statistically different from the one found in WT mice, elacridar greatly increased the BBB permeability to [^3^H]-SN-38 in the cerebellum (4.2-fold, *p* < 0.05), brainstem (4.4-fold, *p* < 0.01), and cerebrum (6.75-fold, *p* < 0.001). This distinct difference between the TKO (Abcb1a(−/−), Abcb1b(−/−), Abcg2(−/−)) group and the elacridar-treated WT group raises the hypothesis that elacridar may act on a third transporter mainly implicated in the efflux of [^3^H]-SN-38 by the cerebrum BBB. This was further confirmed with TKO mice treated with elacridar, which revealed an increase of the Kin of [^3^H]-SN-38 of 3.15-fold (*p* < 0.001), 2.2-fold (*p* < 0.05), and 2.9-fold (*p* < 0.001) in the cerebellum, brainstem, and cerebrum, respectively, in comparison to the non-treated TKO group.

The use of the MRPs inhibitor MK-571 similarly produced a higher BBB permeability to the [^3^H]-SN-38 in all brain structures analyzed, and in both WT and TKO mice. The initial BBB transport of [^3^H]-SN-38 in WT and TKO increased by 3.5- and 2.8-fold in the cerebellum, 4.0- and 1.75-fold in the brainstem, and 3.0- and 2.55-fold in the cerebrum, respectively. This indicates that MRP activity in the efflux of this anticancer drug is relatively the same, regardless of the brain structure in question. The present results demonstrate that P-gp, Bcrp, and Mrp transporters, and potentially other ABC transporters that are inhibited by elacridar, participate in the efflux of [^3^H]-SN-38 at the BBB.

### 3.5. Brain Distribution and Efflux Transport of [^3^H]-SN-38 in DIPG Rats

**Figure 6:**
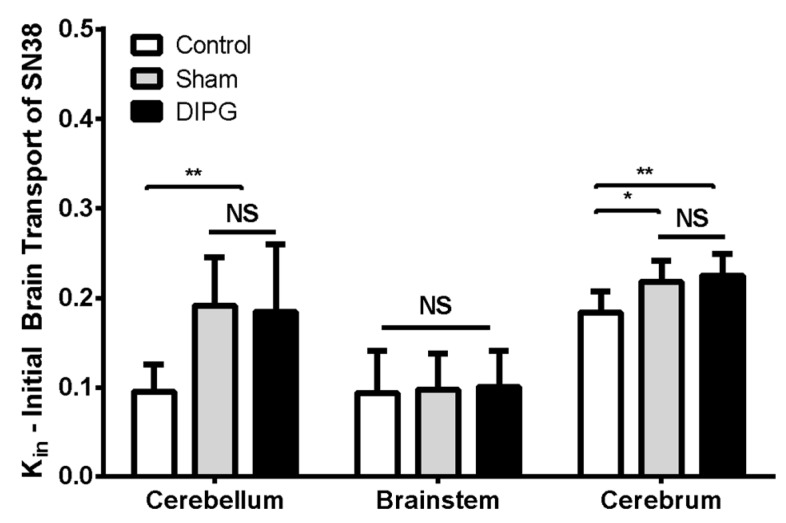
Comparison between control (non-operated), sham, and DIPG cell-derived xenografts of the initial brain transport (Kin; μL s^-1^ g^-1^) of [^3^H]-SN-38 within the cerebellum, brainstem, and cerebrum, measured by in situ brain perfusion using 8-week-old RH-Foxn1rnu rats. Results are expressed as mean ± S.D. (n = 7–10 animals per group). One-way ANOVA, NS: no statistical difference, * *p* < 0.05, ** *p* < 0.01.

The BBB permeability of [^3^H]-SN-38 in RH-Foxn1rnu DIPG-xenografted rats (4-weeks post-implantation) was subsequently analyzed and compared to that of control 8-week-old nude rats either sham-operated or not. In situ brain perfusion of [^3^H]-SN-38 in sham-operated vs. control animals revealed little (Kin, μL s^−1^ g^−1^; cerebellum: 0.19 ± 0.05 in sham vs. 0.10 ± 0.03 in control; cerebrum: 0.22 ± 0.02 in sham vs. 0.18 ± 0.02 in control) to no significant difference (brainstem: 0.10 ± 0.04 in sham vs. 0.09 ± 0.05 in control) on the luminal BBB transport of [^3^H]-SN-38 between groups (Figure 6). This demonstrates that the surgery intervention for DIPG cell implantation had little consequences on the BBB efflux transporter function, where only a slight increase in the initial transport rate of [^3^H]-SN-38 can be seen in the cerebellum and cerebrum. DIPG-bearing animals exhibited very similar [^3^H]-SN-38 Kin to that observed in sham-operated rats (cerebellum: 0.18 ± 0.08, brainstem: 0.10 ± 0.04, cerebrum: 0.22 ± 0.02 μL s^−1^ g^−1^), which is evidence that the tumor growth and infiltration in this DIPG model did not represent any additional damage to the function and integrity of the BBB, nor represented any supplementary restraint to the BBB permeability of SN-38 into the brain.

## 4. Discussion and Conclusions

The chemotherapeutic agent irinotecan and its active metabolite SN-38 show activity in malignant gliomas but have been proven to be clinically ineffective for the treatment of DIPG, with no relevant influence on the response or the event-free survival time [31,32,33]. Such a lack of anticancer activity in vivo may presumably be due to a low penetration of SN-38 into the CNS, notably into the brainstem and cerebellum. The present study focused on understanding how BBB properties and function, in physiological and DIPG conditions, may impact SN-38 BBB luminal transport where DIPG develops. We characterized the transport-mediated drug efflux features of the BBB and the contribution of certain ABC transporters in limiting the brain penetration of this anticancer drug.

Previous studies allowed the generation of an in vivo DIPG model from the injection of tumor cells collected from DIPG patients into the brainstem of immunodeficient animals [34,35,36], as well as with HSJD-DIPG-007 cells used in the present study [11,15]. In the animal models, diffuse infiltrative tumors corresponding to WHO grade III gliomas (anaplastic astrocytoma) grow in the injected area of the pons and invade the cerebellum. We successfully established a cell-derived DIPG rodent model, which at 4 weeks post-tumor cell implantation, consistently produced DIPG-bearing animals exhibiting significant tumor infiltration around the pons and cerebellum, without developing any perceptible clinical signs. The development of such a DIPG animal model allowed us to assess the eventual impact of the developed glioma on the BBB integrity. Our present evaluation of an eventual BBB opening, obtained through analysis of the amount of perfused radiolabeled [^14^C]-sucrose that ultimately would leak from the brain vascular space into the CNS, demonstrated that no further [^14^C]-sucrose permeability occurred in DIPG-bearing animals, in comparison with control animals. In line with these results, the present study also demonstrated that rat DIPG xenografts injected with TRD i.v. evidenced that its brain distribution was restricted to the brain vasculature, in the brainstem, cerebellum, and brain cortex. These results further underline the protective role of the BBB during DIPG invasion and the necessity to develop drugs/strategies able to circumvent it. Some studies indicate that, indeed, a good part of DIPG tumors at diagnosis present a relatively intact and functional BBB when assessed by magnetic resonance imaging (MRI) contrast enhancement [8,37]. A recent preclinical study evidenced a loss of the expression of the glucose transporter GLUT1 (SLC2A1) in the DIPG-engrafted mouse pons, which according to the authors, may indicate a BBB disruption while no Evans blue dye diffusion occurred in the brain, and in particular in the infiltrated pons, when assessing the eventual functional disruption of the BBB in normal and DIPG-bearing mice [38]. However, to our knowledge, a loss of GLUT1 expression is independent of a loss of BBB integrity. Model-specific properties may also affect BBB integrity in DIPG. For instance, a recent study that measured the TRD distribution in the brain of a genetically generated brainstem glioma mouse model evidenced that the structural integrity of the BBB was compromised in the brainstem but not in the cortex of tumor-bearing mice [10]. Our present results evidence that there is no loss of BBB integrity in the framework of the HSJD-DIPG-007 xenograft. Interestingly, previous research conducted in brain gliomas evidenced a significantly higher BBB restriction in brainstem pediatric HGG (pHGG) when compared to cortical pHGG, using genetically engineered mouse models of pHGG [6]. DIPG patients may then conserve an intact and functional BBB, which would explain the failure of chemotherapy against this type of brain glioma.

We then explored the eventual consequences of DIPG on the BBB permeability to SN-38 in different brain regions: Brainstem, cerebellum, and cerebrum, distinctively affected in DIPG, by brain-perfusing DIPG-bearing rats with radiolabeled [^3^H]-SN-38. The results obtained allowed us to observe no change in the initial uptake rate of SN-38 in any of the analyzed brain regions, confirming that the highly maintained BBB tightness greatly contributes to the chemotherapeutic resistance observed in DIPG patients.

Our studies conducted by in situ brain perfusion with radiolabeled [^3^H]-SN-38 evidenced that P-gp or Bcrp inhibition alone was not enough to increase the brain levels of this chemotherapeutic agent in the rat. At least an additive effect of P-gp and Bcrp seems to be present in the efflux transport of SN-38 at the rodent BBB, since the dual inhibition of P-gp and Bcrp with elacridar resulted in a substantial increase in SN-38 levels in the brain, both in rats and mice. A synergistic interaction in restricting the brain penetration of overlapping substrates of P-gp and Bcrp has been previously demonstrated [39,40,41], as in the case of topotecan, another camptothecin [42]. However, in TKO-lacking P-gp and Bcrp (Abcb1a(−/−), Abcb1b(−/−), Abcg2(−/−)) mice, the brain penetration of SN-38 was modestly affected in comparison to WT mice, which contrasts with the findings obtained following the co-perfusion with elacridar. The difference found between these two groups raises the hypothesis that elacridar, nowadays known to exclusively inhibit P-gp and Bcrp efflux transporter, might in fact inhibit at least a third efflux transporter, also involved in the efflux of SN-38 at the BBB. The expression of such a transporter should be limited in the cerebellum and in the brainstem, given there was no significant difference between the TKO and elacridar-treated groups in these brain areas, but considerably important within the brain cortex, since a substantial increase in the permeability of SN-38 in elacridar-treated animals was observed in comparison to TKO.

Our observation that the MRP inhibitor MK-571 greatly contributed to an increase of the SN-38 permeability across the mouse BBB suggests that this ABC efflux transporter plays an important role in the brain-to-blood transport of camptothecins like SN-38, even though MRPs are far from representing the major efflux transporters present at the BBB [43,44]. Earlier studies have already evidenced that irinotecan and its active metabolite, SN-38, are substrates of ABC efflux transporters expressed in different tissues [45,46,47,48], including the BBB [49,50]. Brain-to-plasma ratios of irinotecan and SN-38 have been shown to be higher in mice lacking P-gp [49]. In line with our results, a previous study demonstrated that P-gp, Bcrp, and Mrp4 participate in the brain efflux of both irinotecan and SN-38, as their triple deficiency in mice increased the brain concentrations of irinotecan and SN-38 15-fold and 6.6-fold, respectively [50]. In this study, the additional deficiency of Abcc4/Mrp4 in Abcb1a/b, Abcg2(−/−, −/−) mice represented a further increase of the brain levels of irinotecan and SN-38 by 1.6-fold and 5.8-fold, respectively [50], evidencing that SN-38 was likely an excellent Mrp4 substrate, which is in agreement with our MRPs inhibition findings with MK571.

In agreement with these functional results, targeted proteomics of the major ABC-transported P-gp, BCRP, and MRP1 in the whole tissue evidenced that there is unfortunately no downregulation of the basal expression of these efflux transporters in DIPG-bearing animals. This further indicates that such transporters are present and readily active at the BBB in the DIPG condition, and so available to efflux their drug substrates. Furthermore, the absence of any difference in the SN-38 BBB permeability observed between DIPG-bearing and control animals is also in line with the absence of any variation in the expression of SN-38 transporters in the DIPG condition at the luminal BBB. Although an opposite regulation with both up- and downregulation of SN-38 transporters, which functionally compensate, could not be excluded, our proteomic analysis does not support such a hypothesis. However, the targeted proteomic analysis performed from whole homogenates and not from isolated brain capillaries (the latter proven difficult to perform given the small size of each brain structure) from each brain region limits our conclusions regarding more subtle variations in the expression of these transporters at the BBB, namely for MRP4, whose expression was below the LOQ. As also suggested by our targeted proteomic analysis, DIPG cells also express BCRP, which should represent a second “barrier” to the delivery of anticancer drugs. Nonetheless, the fact that DIPG cells do not express P-gp, MRP1, or MRP4 suggests a low drug efflux capacity at this level, and consequently, SN-38 is likely to reach its cellular target once it has crossed the BBB.

In conclusion, this present study provides further and solid evidence that the BBB remains tight and functionally active in a clinically relevant DIPG model, contributing to the lack of effectiveness of anticancer drugs against this type of glioma. Therefore, in DIPG and up to the later stages of the disease, the maintenance of the paracellular route at the BBB is preserved as well as a conserved function of SN-38 transporters at the brain endothelial cell level. In fact, the major ABC transporters present at the human and rodent BBB—P-gp, BCRP, but also MRPs and other efflux transporter(s) that are elacridar sensitive and not yet identified—were here shown to be implicated in the efflux of SN-38, preventing its chemotherapeutic action. Thus, our present study further underlines the need for the development of new strategies to improve the brain drug delivery of chemotherapeutic agents, such as that of SN-38, in order to overcome the BBB and possibly ABC efflux at both BBB and DIPG membranes to gain a more effective cytotoxic cell concentration.

## Figures and Tables

**Table 1:** Expression of selected ABC transporters in HSJD-DIPG-007 cells, measured by HPLC-MS/MS. Results are expressed as the mean absolute concentration (fmol μg^−1^) of duplicate measurements (*n* = 2).

Protein Expression (fmol μg^−1^)	P-gp	BCRP	MRP1	MRP4	Nestin
HSJD-DIPG-007 sample 1	<LOQ	0.94	<LOQ	<LOQ	3.30
HSJD-DIPG-007 sample 2	<LOQ	2.55	<LOQ	<LOQ	3.23

## Supplementary Materials

The following are available online at https://www.mdpi.com/1999-4923/12/5/399/s1, Figure S1: Brain vascular space (A) and initial brain transport (Kin; μL s^-1^ g^-1^) of [^3^H]-SN-38 (B) within the cerebellum, brainstem, and cerebrum, in 4-week-old RH-Foxn1rnu male and female nude rats, Table S1: Target peptides and selected ions used in the UHPLC-MS/MS multiplexed SRM method analysis.
