# Supplementary material for: Characterization of the Blood–Brain Barrier Integrity and the Brain Transport of SN-38 in an Orthotopic Xenograft Rat Model of Diffuse Intrinsic Pontine Glioma
Source: Pharmaceutics. 2020 Apr 27;12(5):399. doi: 10.3390/pharmaceutics12050399 (PMC7284501; doi:10.3390/pharmaceutics12050399)
Supplement: Supplementary file 1 [file pharmaceutics-12-00399-s001.pdf]

pharmaceutics

Supplementary Materials: Characterization of the Blood-Brain Barrier Integrity and the Brain Transport of SN-38 in an Orthotopic Xenograft Rat Model of Diffuse Intrinsic Pontine Glioma

Catarina Chaves, Xavier Declèves, Meryam Taghi, Marie-Claude Menet, Joelle Lacombe, Pascale Varlet, Nagore G. Olaciregui, Angel M. Carcaboso, and Salvatore Cisternino

Comparison of the brain distribution of [^{14}C]-sucrose (vascular volume and integrity marker) and [^{3}H]-SN-38 in male and female RH-Foxn1rnu nude rats

In order to assess whether a gender-difference is observed in what concerns to the brain vasculature and distribution of [^{3}H]-SN-38, athymic RH-Foxn1rnu male and female nude rats (n = 5 per group, 4-week-old) were brain perfused in situ with radiolabeled [^{3}H]-SN-38 and [^{14}C]-sucrose. The initial brain transport rate for [^{3}H]-SN-38 was calculated taking into account the brain vascular volume, which is given by the local distribution of sucrose, confined to the vascular space within the brain. This study demonstrated that no significant difference (NS) between genders was observed for the Kin of [^{3}H]-SN-38 in any of the analyzed brain regions (p > 0.05, Supplementary Figure S1).

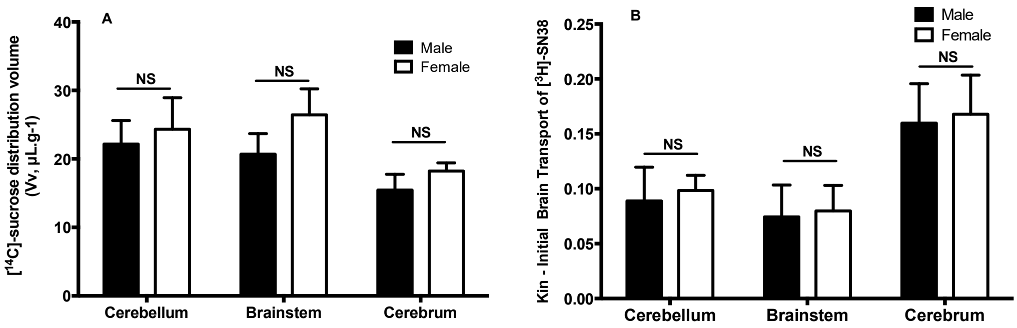

Figure S1. Brain vascular space (**A**) and initial brain transport (Kin; µL·s^{-1}·g^{-1}) of [^{3}H]-SN-38 (B) within the cerebellum, brainstem, and cerebrum, in 4-week-old RH-Foxn1rnu male and female nude rats. Results are expressed as mean Kin ± S.D. (n = 5 animals per group). Student-t test, NS p > 0.05 female vs male group.

Table S1. Target peptides and selected ions used in the UHPLC-MS/MS multiplexed SRM method analysis.

Protein	Species Specificity	Peptide Sequence	Isotope Type	Precursor M/z	Precursor Charge	Product M/z	Product Charge	Fragment Ion Type
BCRP (ABCG2)	Human, Mouse, Rat	SSLLDVLAAR	light	522.8	2	757.5	1	y7
						644.4	1	y6
						529.3	1	y5
						430.3	1	y4
		SSLLDVLAAR	heavy	524.8	2	761.5	1	y7
						648.4	1	y6
						533.4	1	y5
						434.3	1	y4
P-gp (ABCB1)	Human, Mouse, Rat	LANDAAQVK	light	465.3	2	816.4	1	y8
						745.4	1	y7
						631.3	1	y6
						516.3	1	y5
		LANDAAQVK	heavy	468.3	2	822.4	1	y8
						751.4	1	y7
						637.4	1	y6
						522.3	1	y5

MRP1 (ABCC1)	Human, Rat	TPSGNLVNR	Light	479.3	2	759.4	1	y7
						672.4	1	y6
						615.4	1	y5
						501.3	1	y4
		TPSGNLVNR	Heavy	482.8	2	766.4	1	y7
						679.4	1	y6
						622.4	1	y5
						508.3	1	y4
MRP4 (ABCC4)	Human, Rat	APVLFFDR	Light	482.8	2	786.4	1	y6
						697.4	1	y5
						584.3	1	y4
						537.2	1	y3
		APVLFFDR	Heavy	486.3	2	803.5	1	y6
						704.4	1	y5
						584.3	1	y4
						437.2	1	y3
NESTIN	Human	GLVEGASVK	Light	430.2	2	802.5		y8
						689.4		y7
						590.3		y6
						461.3		y5
		GLVEGASVK	Heavy	442.3	2	826.5		y8
						713.4		y7
						608.3		y6
						479.3		y5
